# Importância Diagnóstica e Prognóstica da Capacidade Funcional nas Diversas Formas Evolutivas da Doença De Chagas

**DOI:** 10.36660/abc.20210808

**Published:** 2021-11-01

**Affiliations:** 1 Universidade de São Paulo Faculdade de Medicina Instituto do Coração do Hospital das Clínicas São Paulo SP Brasil Instituto do Coração do Hospital das Clínicas da Faculdade de Medicina da Universidade de São Paulo, São Paulo, SP – Brasil; 2 Universidade do Estado do Amazonas Manaus AM Brasil Universidade do Estado do Amazonas, Manaus, AM – Brasil

**Keywords:** Doença de Chagas, Exercício, Cardiopatia Chagasica, Teste de Esforço/métodos, Insuficiência Cardíaca/complicações, Tromboembolia, Trypanosoma Cruzi

O estudo publicado por Silva et al.,^1^ nesta edição nos traz uma reflexão importante sobre a evolução das diversas formas da doença de Chagas (DC), ao demonstrar que os pacientes com a forma indeterminada (FI) possuem capacidade funcional, medida através do consumo de oxigênio de pico (VO_2_pico), semelhante a indivíduos saudáveis sem DC. Por outro lado, pacientes com cardiopatia chagásica sem disfunção ventricular apresentaram capacidade funcional semelhante aos pacientes com disfunção ventricular.^1^

A FI da DC vem sendo estudada sob diversos aspectos há vários anos. Alguns estudos em pacientes assintomáticos e que se incluem na definição da FI por não apresentarem alterações eletrocardiográficas e na radiografia de tórax, demonstraram alterações incipientes em exames complementares que podem sugerir a possibiliade de evolução para as formas mais graves de DC ao longo dos anos. Avaliações feitas através da ecocardiografia apresentaram alterações em variáveis como Doppler tecidual e estudo da deformidade miocárdica através do *strain* bidimensional.^2,3^ Já houve demonstração também de alterações do sistema nervoso autônomo, principalmente no ramo parassimpático, que podem ser potenciais vias de piora no estágio clínico da doença no decorrer dos anos.^4,5^ Estudos feitos com ressonância magnética demonstraram presença de fibrose miocárdica em 12% de pacientes com a FI da doença.^6^ No entanto, apesar dessas pequenas alterações, a evolução a longo prazo desses pacientes tem se mostrado favorável e semelhante à de indivíduos saudáveis sem DC. Ianni et al.,^7^ estudaram pacientes com a FI com base nos achados do eletrocardiograma (ECG) por 8 anos e concluíram que a FI da DC representa uma condição benigna com prognóstico favorável em longo prazo.^7^ No entanto, em um pequeno grupo de pacientes pode haver evolução para cardiopatia chagásica crônica (CCC) ou doença do aparelho digestivo em cerca de 10 a 20 anos após a infecção aguda. Sabino et al.,^8^ em um estudo de coorte retrospectivo de 10 anos, sugeriram uma taxa de progressão para cardiomiopatia de 1,85% ao ano em pacientes com a FI da doença.^8^ Portanto, são necessários estudos que identifiquem marcadores que possam predizer a possibilidade desta evolução e a avaliação da capacidade funcional destes pacientes é importante neste aspecto.

A presença de alterações eletrocardiográficas sugestivas de comprometimento cardíaco, características da DC, em um indivíduo sintomático ou assintomático, caracteriza a forma cardíaca crônica da DC. Este grupo de pacientes pode se apresentar apenas com o ECG alterado, porém sem sintomas ou presença de disfunção ventricular ou apresentar-se com sintomas de insuficiência cardíaca e disfunção sistólica ventricular esquerda de grau até importante. Estudos com ressonância magnética demonstraram presença de até 94% de fibrose miocárdica em pacientes com ECG alterado, mesmo sem disfunção ventricular.^6^ Estes achados sugerem que este grupo de pacientes, deve ter um acompanhamento clínico rigoroso e a avaliação da capacidade funcional também se enquadra neste espectro.

Por outro lado, pacientes com CCC e grave disfunção ventricular representam um grupo de pacientes que têm um prognóstico pior do que outras etiologias de miocardiopatias. Mady et al.,^9^ demonstraram que a capacidade funcional, assim como a fração de ejeção, é um importante preditor de sobrevida neste grupo de pacientes.^9^ Além disto, o treinamento físico e a reabilitação cardíaca são importantes componentes de melhora clínica destes pacientes e o VO_2_pico é também importante nesta monitorização.^10^

Todos estes aspectos sugerem que o estudo da DC necessita abordar cada vez mais temas que busquem preditores de sua evolução tão variável de indivíduo para indivíduo e o estudo da capacidade funcional é importante neste contexto.
