# Supplementary material for: Importância Diagnóstica e Prognóstica da Capacidade Funcional nas Diversas Formas Evolutivas da Doença De Chagas
Source: Arq Bras Cardiol. 2021 Nov 1;117(5):942–3. [Article in Portuguese] doi: 10.36660/abc.20210808 (PMC8682086; doi:10.36660/abc.20210808)
Supplement: Translation

# Translation: Diagnostic and Prognostic Importance of Functional Capacity in the Different Evolutionary Forms of Chagas Disease

**Affiliations:** 1 São Paulo SP Brazil Instituto do Coração do Hospital das Clínicas da Faculdade de Medicina da Universidade de São Paulo, São Paulo, SP – Brazil; 2 Manaus AM Brazil Universidade do Estado do Amazonas, Manaus, AM – Brazil

**Keywords:** Chagas Disease, Exercise, Chagas cardiomyopathy, Exercise Test/methods, Heart Failure/complications, Thromboembolismi, Trypanosoma Cruzi

The manuscript published by Silva et al.^1^ in this issue brings us an important reflection on the evolution of the different forms of Chagas disease (CD), by demonstrating that patients with the indeterminate form (IF) have similar functional capacity, measured through peak oxygen uptake (VO_2_peak), to healthy individuals without CD. On the other hand, patients with Chagas’ heart disease without ventricular dysfunction had similar functional capacity to patients with ventricular dysfunction.^1^

The IF of CD has been studied under different aspects for several years. Some studies in asymptomatic patients that are included in the definition of IF for not presenting electrocardiographic and chest X-ray alterations, demonstrated incipient alterations in complementary exams that may suggest the possibility of evolution to the more severe forms of CD over the years. Evaluations made through echocardiography showed alterations in variables such as tissue Doppler and study of myocardial deformity through two-dimensional strain.^2,3^ There has also been demonstration of changes in the Autonomic Nervous System, especially in the parasympathetic branch, which can be potential pathways for worsening in the stage disease over the years.^4,5^ Studies performed with magnetic resonance have demonstrated the presence of myocardial fibrosis in 12% of patients with IF of the disease.^6^ However, despite these small changes, the long-term evolution of these patients has been shown favorable and similar to that of healthy individuals without CD. Ianni et al.^7^ studied patients with the IF based on ECG findings for 8 years and concluded that the IF of CD represents a benign condition with a favorable long-term prognosis.^7^ However, in a small group of patients, there may be evolution for chronic Chagas cardiopathy (CCC) or digestive tract disease in about 10 to 20 years after acute infection. Sabino et al.,^8^ in a 10-year retrospective cohort study, suggested a rate of progression to cardiomyopathy of 1.85% per year in patients with FI of the disease.^8^ Therefore, studies that identify markers that can predict the possibility of this evolution are needed and the evaluation of the functional capacity of these patients is important in this aspect.

The presence of electrocardiographic alterations suggestive of cardiac involvement, characteristic of CD, in a symptomatic or asymptomatic individual, characterizes the chronic cardiac form of CD. This group of patients may present only with an altered electrocardiogram (ECG), but without symptoms or presence of ventricular dysfunction, or present with symptoms of heart failure and significant grade of left ventricular systolic dysfunction. Studies with magnetic resonance have shown the presence of up to 94% of myocardial fibrosis in patients with altered ECG, even without ventricular dysfunction.^6^ These findings suggest that this group of patients should have a rigorous clinical follow-up and the assessment of functional capacity is also important into this spectrum.

On the other hand, patients with CCC and severe ventricular dysfunction represent a group of patients who have a worse prognosis than other cardiomyopathy etiologies. Mady et al.^9^ demonstrated that functional capacity, as well as ejection fraction, is an important predictor of survival in this group of patients.^9^ In addition, physical training and cardiac rehabilitation are important components of clinical improvement in these patients and VO_2_peak is also important in this monitoring.^10^

All these aspects suggest that the study of CD needs to increasingly address themes that seek predictors of its evolution, which varies from individual to individual, and the study of functional capacity is important in this context.
